# Effect of Vacancy Defects on the Vibration Frequency of Graphene Nanoribbons

**DOI:** 10.3390/nano12050764

**Published:** 2022-02-24

**Authors:** Hong Guo, Jing Wang

**Affiliations:** Xinjiang Key Laboratory of Solid State Physics and Devices, College of Physics Science and Technology, Xinjiang University, Urumqi 830046, China; gh530468@163.com

**Keywords:** graphene nanoribbons, vacancy defect, vibration frequency, molecular dynamics simulation

## Abstract

Graphene is a type of two-dimensional material with special properties and complex mechanical behavior. In the process of growth or processing, graphene inevitably has various defects, which greatly influence the mechanical properties of graphene. In this paper, the mechanical properties of ideal monolayer graphene nanoribbons and monolayer graphene nanoribbons with vacancy defects were simulated using the molecular dynamics method. The effect of different defect concentrations and defect positions on the vibration frequency of nanoribbons was investigated, respectively. The results show that the vacancy defect decreases the vibration frequency of the graphene nanoribbon. The vacancy concentration and vacancy position have a certain effect on the vibration frequency of graphene nanoribbons. The vibration frequency not only decreases significantly with the increase of nanoribbon length but also with the increase of vacancy concentration. As the vacancy concentration is constant, the vacancy position has a certain effect on the vibration frequency of graphene nanoribbons. For nanoribbons with similar dispersed vacancy, the trend of vibration frequency variation is similar.

## 1. Introduction

Graphene has excellent elastic properties and high intrinsic strength [1,2,3,4], as well as extraordinary mechanical [5,6,7], electrical [8,9], and other physical [10,11,12] properties, making it one of the most promising materials in modern technology. Therefore, graphene is widely used in new energy research fields such as nanosensors [13,14,15], composite materials [16], drug delivery agents [17], solar cells, and supercapacitors [18,19], and also has an irreplaceable position in many fields, thus attracting much attention. It is clear that these properties depend on the structure of the hexagonal graphene lattice and the strong in-plane *sp*^2^ bonds between the carbon atoms. The mechanical property of graphene is crucial for its reliable application, both from a technical point of view and from the perspective of understanding its fundamental interest in physics deformation [20,21,22]. However, in the actual preparation of graphene, it is almost impossible to obtain perfectly structured graphene, and some defects such as Stone–Thrower–Wales (STW) defects [23], vacancy defects [24], grain boundaries [25], and adsorbed atoms [26] are inevitable. The most common defects are vacancy defects. The structural symmetry and integrity of graphene can be reduced due to the presence of lattice defects, which changes the property of graphene. Due to the presence of vacancy defects, the tensile strength of graphene is reduced [27]. Wang et al. [28] found that both Stone–Thrower–Wales and vacancy defects can lead to a significant reduction in the mechanical strength of graphene. Rahmandoust et al. [29] investigated dislocation, vacancy, and Stone–Wales defects in the semiconductor graphene quantum dot model, showing that mechanical property reduction is close to the experimentally measured values, while the increase in the number of layers does not significantly affect the final results. Park et al. [30] investigated the effect of defect size on the mechanical properties of graphene, in which large-sized vacancy defects have a higher tensile modulus and lower fracture strain compared to small-sized vacancy defects. Motamedi et al. [31] studied the effect of central cracking on graphene, and Young’s modulus of graphene decreased from 905 GPa to 697 GPa as the crack length increased. Lopez-Polin et al. [32] conducted a systematic study on the elastic modulus and strength of graphene with controllable defect density; the results show that Young’s modulus increases with increasing defect density, elastic modulus decreases with defect inclusions for higher density defects, and fracture strength decreases with increasing defect density. Ng et al. [33,34] used molecular dynamics (MD) simulations to study the change in thermal conductivity of graphene under two different chiralities and the relationship between chirality and STW defect density. The results showed that the thermal conductivity decreased with increasing STW defect density, independent of chirality. Lee et al. [35] measured the elastic properties and intrinsic fracture strength of freestanding monolayer graphene films using nanoindentation and showed that atomically perfect nanoscale materials could be tested for deformation using mechanical methods over a range well beyond the linear range. Therefore, the introduction of defects has an important effect on the structural properties of graphene. In this paper, we use molecular dynamics simulations to investigate the effects of different defect concentrations and defect locations on the vibration frequency of monolayer graphene.

## 2. Physical Model and Simulation Method

In this paper, the vibration frequencies of monolayer graphene nanoribbons with different defect concentrations and different defect locations were calculated using the Forcite Tools module of MS (Materials Studio 8.0) software and the molecular dynamics method. It is predictable that ideal graphene nanoribbons and graphene nanoribbons with different vacancy defects produce different vibrations under the same simulation conditions. The structure of graphene nanoribbons was fixed with the width (3.935 nm) and the thickness (2.0 nm), and the length increased from 8.379 nm to 23.718 nm.

**Figure 1:**
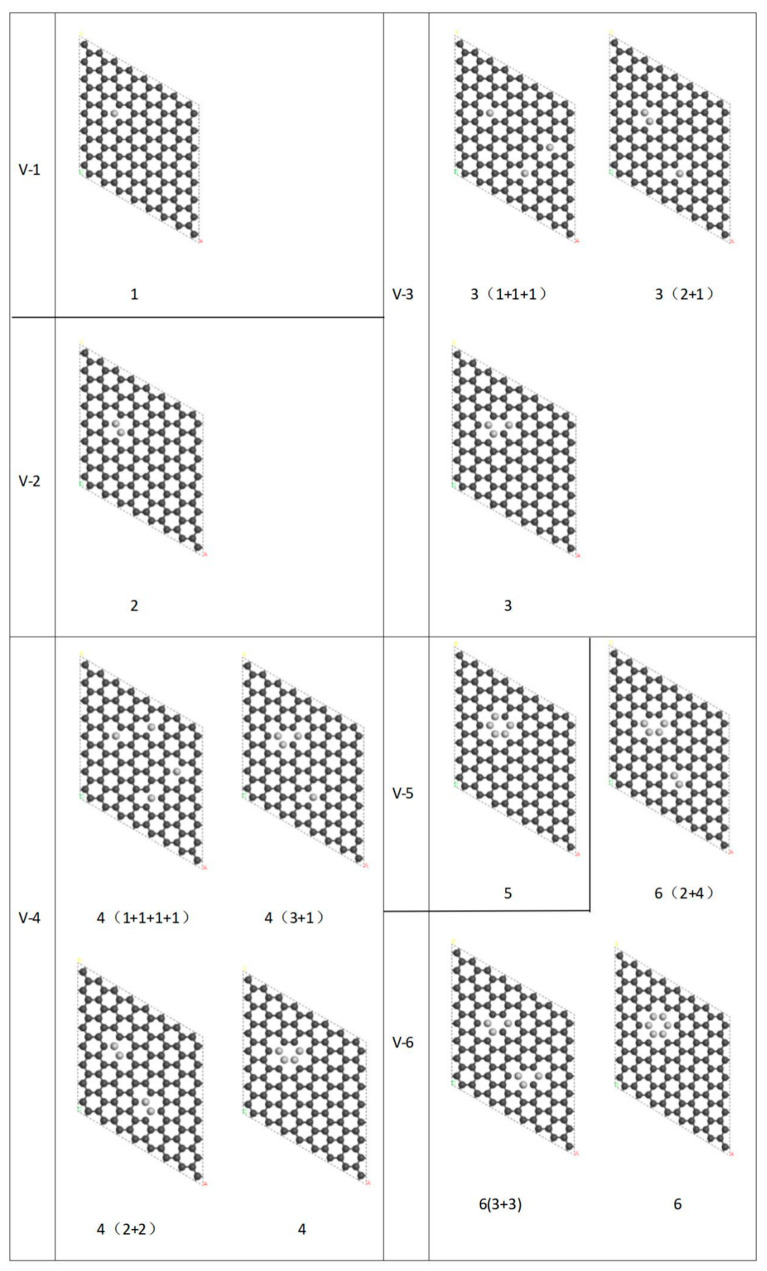
Types of vacancy defect in a supercell of graphene. The dark spheres represent carbon atoms and the light spheres represent missing carbon atoms.

**Figure 2:**
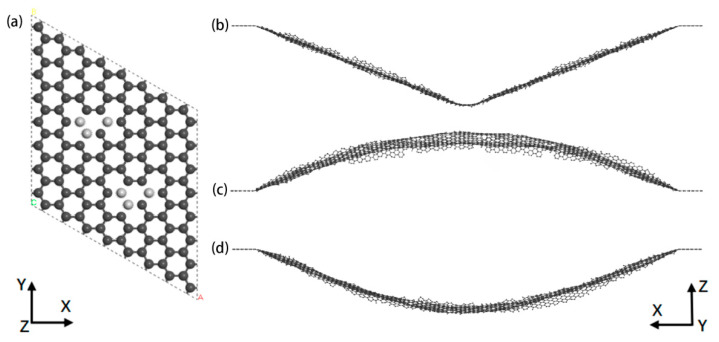
Graphene nanoribbons (**a**) Graphene supercell containing vacant 6(3 + 3) sites; (**b**) Initial bending state; (**c**,**d**) are vibration states at a certain time.

**Figure 3:**
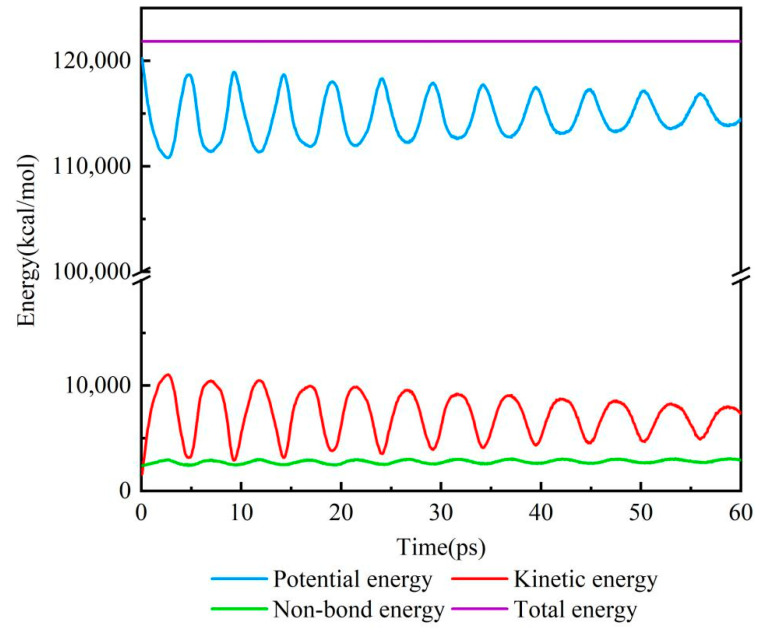
Energy of graphene nanoribbons during vibration.

The types of vacancy defects in graphene we discuss in this paper (based on a supercell with the number of atoms 8 × 8 × 1) are shown in Figure 1, where the dark spheres represent carbon atoms and the light spheres represent missing atoms. V-*i* (*i* = 1, 2, ……, 6) indicates the type of vacancy and *i* is the number of missing atoms in a supercell. For example, 3(2 + 1) indicates that the number of missing atoms in the supercell is three, and (2 + 1) indicates the combination of missing atoms. The structure of graphene nanoribbons was obtained by expanding the supercells of the above model according to the requirement. Figure 2a show a graphene supercell containing a vacancy type of 6(3 + 3). A silicon probe was placed directly on top of the graphene nanoribbon, which is close to the nanoribbon but does not bind to the atoms on the surface of the graphene. The structurally optimized nanoribbons bent due to the presence of Van der Waals forces between the silicon probe and the graphene nanoribbons [36,37]. Subsequently, the silicon probe was removed as the nanoribbon was bent to a certain extent, which is the initial bending state of the graphene nanoribbon shown in Figure 2b. Then, dynamic mechanical simulations of bent nanoribbons were performed in the NVE ensemble. Figure 2c,d show the vibration of the graphene nanoribbon at a certain time, respectively. The COMPASS II force field [38] was selected for all structure optimization during the simulation, the simulation temperature was 300 K and the truncation radius was 15.5 Å. As the length of the graphene nanoribbon increased, the vibrational period of its energy also increased; therefore, the simulation time was set to 60 ps with a time step of 1 fs [39]. The total energy of the nanoribbon was obtained by simulation, as shown in Figure 3, and the kinetic energy and the potential energy fluctuated during the vibration of the nanoribbon. Kinetic energy and potential energy are converted into each other, and the vibration period can be obtained according to the curve of kinetic energy and potential energy, from which the vibration frequency of the graphene nanoribbons can also be obtained.

## 3. Results and Discussion

The structure of different vacancy supercells was expanded to study the effect of the vacancy concentration of graphene nanoribbons with different lengths on their vibration frequencies. As the vacancy concentration was certain, the effect of the vacancy position of graphene nanoribbons on their vibration frequency was also explored.

**Figure 4:**
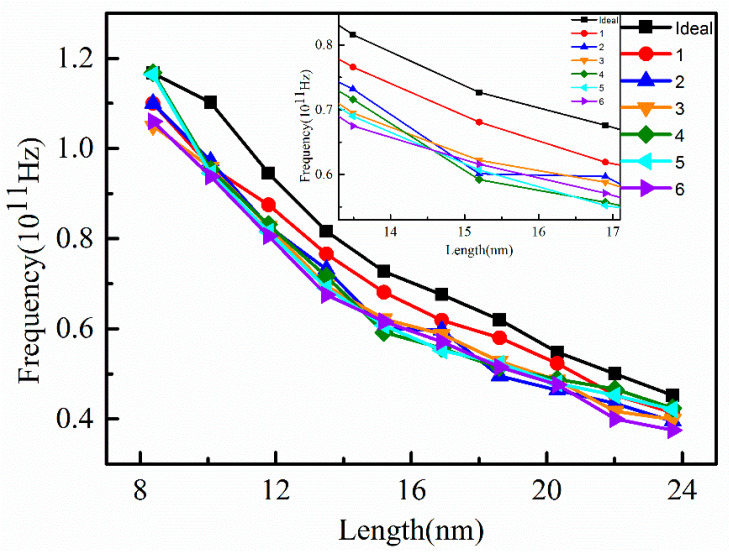
Vibration frequency with length of graphene nanoribbons with different vacancy concentrations.

The vibration frequency of graphene nanoribbons with different vacancy concentrations were obtained from the energy of the simulation results using molecular dynamics method, as shown in Figure 4. It can be seen that the vibration frequency of the vacancy-free graphene nanoribbon (Ideal) decreased monotonically as the length of the graphene nanoribbon increased. The existence of vacancy defects leads to significant changes in the structure of carbon atoms around the vacancies, as well as in the bond-to-bond strength and bonding mode. Taking the single-vacancy supercell (V-1) (shown in Figure 1), for example, the length of the nearest C-C bond of the ideal graphene nanoribbon was 1.403 Å, the distance of the second nearest neighbor C atom was 2.327 Å, the distance of the third nearest neighbor C atom was 2.764 Å, and the adjacent C-C-C bond angle was 122.984°. However, in the nanoribbon with vacancy, the length of the C-C bond was 1.483 Å, the distance of the second nearest neighbor C atom was 2.430 Å, the distance of the third nearest neighbor C atom was 3.015 Å, and the bond angle between adjacent atoms was 126.616°. This comparison means that the positions of carbon atoms in the nanoribbons changed. That is to say, the bond lengths and bond angles between C atoms were significantly changed. Due to the existence of vacancy defects, the bond lengths and bond angles generally increased after structure optimization, which led to the structural change of the carbon atoms around the vacancies, thus affecting the structure of the nanoribbons.

Therefore, the variation of bond length and bond angle mades the C-C bond energy of graphene vancancy-containing nanoribbons smaller than the ideal structure, which meant that the graphene nanoribbons containing vacancies acquired less deformation energy than that of the ideal graphene nanoribbons after bending. In addition, the deformation energy of graphene nanoribbons with multiple vacancies was converted to smaller vibration energy in the NVE system synthesis. Since the vibration energy was proportional to the square of the vibration frequency, the vibration frequency of graphene nanoribbons containing vacancies was less than that of ideal graphene nanoribbons.

In contrast, the inset shows that the vibration frequency of graphene nanoribbons containing different vacancy concentrations decreased with increasing length as the nanoribbon length increased from 13 nm to about 17 nm. In particular, the vibration frequency of the graphene nanoribbons containing five vacancies decreased sharply; the reduction was greater than that of other vacancies. When the length exceeded 20 nm, the reduction in the vibration frequency changed slowly. The vibration frequencies of the nanoribbons containing single vacancies were significantly higher than those of other vacancies, mainly because the interactions between atoms around the vacancies changed little. In addition, the bond lengths and bond angles between the carbon atoms in nanoribbons containing single vacancies varied less, which had less influence on the vibration frequency.

Except for a single vacancy, the decreasing trend of vibration frequency of other vacancies was not obvious. In general, compared with ideal graphene nanoribbons, the vibration frequency of nanoribbons containing vacancies decreased with increasing vacancy concentration. Therefore, vacancy concentration has a certain effect on the vibration frequency of nanoribbons.

**Figure 5:**
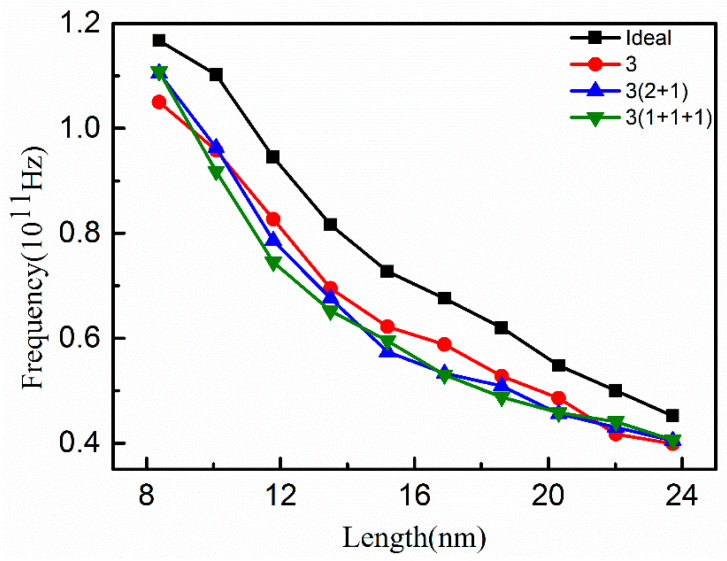
Variation of vibration frequency of graphene nanoribbons at different vacancy positions (V-3) with length.

In addition, dynamic simulation of graphene nanoribbons with different vacancy positions was carried out while the vacancy concentration was kept constant to further understand the influence of different vacancy positions on the vibration frequency of nanoribbons. As shown in Figure 5, it can be seen that the vibration frequency of the graphene nanoribbons without vacancy (ideal) and with a certain vacancy concentration but different vacancy positions (V-3) both decreased monotonically with the increase in length of the graphene nanoribbons. There was a significant difference in the vibration frequency between the ideal and the vacancy nanoribbon. The vibration frequency of graphene nanoribbons was not significantly affected by different vacancy positions at the same vacancy concentration. When the nanoribbon length was less than 10 nm, the vibration frequencies of the dispersed vacancies 3(2 + 1) and 3(1 + 1 + 1) were greater than that of the concentrated vacancies 3. Especially in the length range of 10–20 nm, the vibration frequency of graphene nanoribbons with dispersed vacancy was generally smaller than that of the vacancy concentrated nanoribbons, indicating that the vacancy location had a certain influence on the vibration frequency of graphene nanoribbons. As the nanoribbon length became larger than 20 nm, the influence became insignificant.

**Figure 6:**
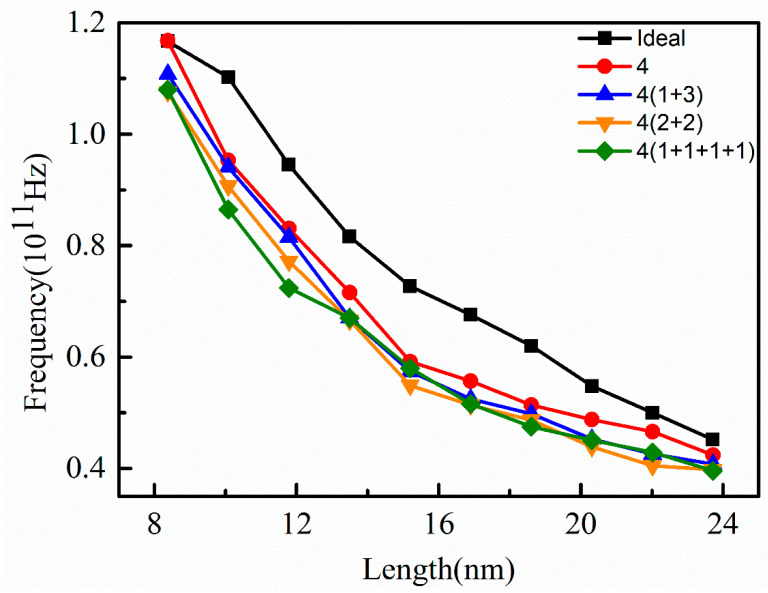
Variation of vibration frequency of graphene nanoribbon at different vacancy positions (V-4) with length.

As the number of the vacancy in a supercell was four (V-4), the effect of different vacancy positions on the vibration frequency of graphene nanoribbons with (V-4) were discussed, as shown in Figure 6. When the nanoribbon length was about 8.5 nm, the vibration frequency of the graphene nanoribbons with concentrated vacancies was identical to that of the ideal graphene nanoribbon, indicating that the concentrated vacancies had less effect on the vibration frequencies of the nanoribbon with this length. When the length of the nanoribbon increased from 8.5 nm to about 13.5 nm, the vibration frequency of nanoribbons with concentrated vacancies and dispersed vacancies decreased sharply, and the decrease of vibration frequency of dispersed vacancies was smaller than that of concentrated vacancies. As the nanoribbons were longer than 15 nm, the decrease of the vibration frequency of nanoribbons with concentrated and dispersed vacancies decreased, and the vibration frequencies of nanoribbons with dispersed vacancies at different positions were almost the same, which indicated that the position of vacancies had little effect on the vibration frequency. By comparing Figure 5 and Figure 6, it can be found that different vacancy positions have a certain influence on the vibration frequency of the nanoribbon. In general, the effect of concentrated vacancies is small, while that of dispersed vacancies is large. When the combination of the vacancy is similar, such as 4(2 + 2) and 4(1 + 3), the vibration frequencies of the nanoribbon are almost the same.

**Figure 7:**
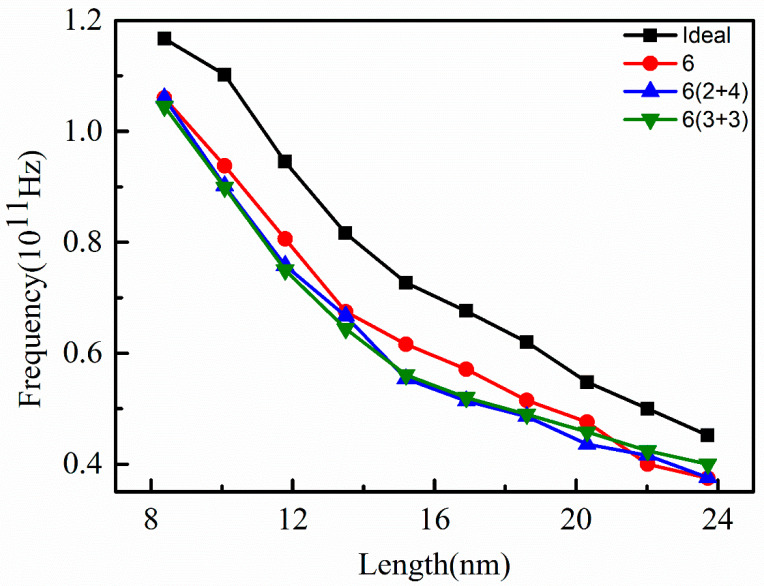
Variation of vibration frequency of graphene nanoribbon at different vacancy positions (V-6) with length.

Compared with other vacancies, the vibration frequency of graphene nanoribbons containing vacancies (V-6) is significantly smaller than that without vacancies (Ideal) when the nanoribbon length is small, as shown in Figure 7. When the nanoribbon lengths were 8.5 nm and 13.5 nm, the effect of concentrated vacancies and dispersed vacancies on the vibration frequency was not much different as the vacancy concentration was certain. When the length of the nanoribbon was between 13.5 nm and 20 nm, the vibration frequency of the nanoribbon with dispersed vacancies was smaller than that of concentrated vacancies, which is mainly related to the bonding mode and strength of atoms around the vacancy. As the length was larger than 20 nm, the vibration frequency was almost independent of the vacancy position with the increase of nanoribbon length. In general, as the combination of the vacancy was similar, the vibration frequencies of the nanoribbon were almost the same, such as 6(2 + 4) and 6(3 + 3), which coincided with that of 4(1 + 3) and 4(2 + 2), shown in Figure 6. This means that the more similar the vacancy positions, the closer the vibration frequency of graphene nanoribbons.

## 4. Conclusions

Defects are inevitable in the actual preparation and processing of graphene. The effects of vacancy defect concentrations and vacancy positions on the vibration frequency of monolayer graphene were studied using molecular dynamics simulations. The results show that vacancy defects reduce the vibration frequency of the graphene nanoribbon, and both vacancy concentration and vacancy position have an effect on the vibration frequency of graphene nanoribbons. Regardless of the presence of vacancy defects, the vibration frequency of graphene nanoribbons decreases with the increase of length. Compared with ideal nanoribbons, nanoribbons with vacancies have smaller vibration frequencies. The vibration frequency of nanoribbons decreases with increasing vacancy concentration, especially when the length of the nanoribbon is small.

Furthermore, the vibration frequency of graphene nanoribbons with different vacancy positions showed little difference. The vibration frequency of the nanoribbon with dispersed vacancy was less than that of the nanoribbon with concentrated vacancy. As the vacancy positions were similar, the vibration frequency of graphene nanoribbons was close. In conclusion, both vacancy concentration and vacancy position have a certain effect on the vibration frequency of graphene nanoribbons, but the size effect of nanoribbons is the main factor.

## Data Availability

The data presented in this study are available on request from the corresponding author.
